# Supplementary material for: Carvedilol targets β-arrestins to rewire innate immunity and improve oncolytic adenoviral therapy
Source: Commun Biol. 2022 Feb 3;5:106. doi: 10.1038/s42003-022-03041-4 (PMC8813932; doi:10.1038/s42003-022-03041-4)
Supplement: Supplementary file 2 — Supplementary Figures [file 42003_2022_3041_MOESM2_ESM.pdf]

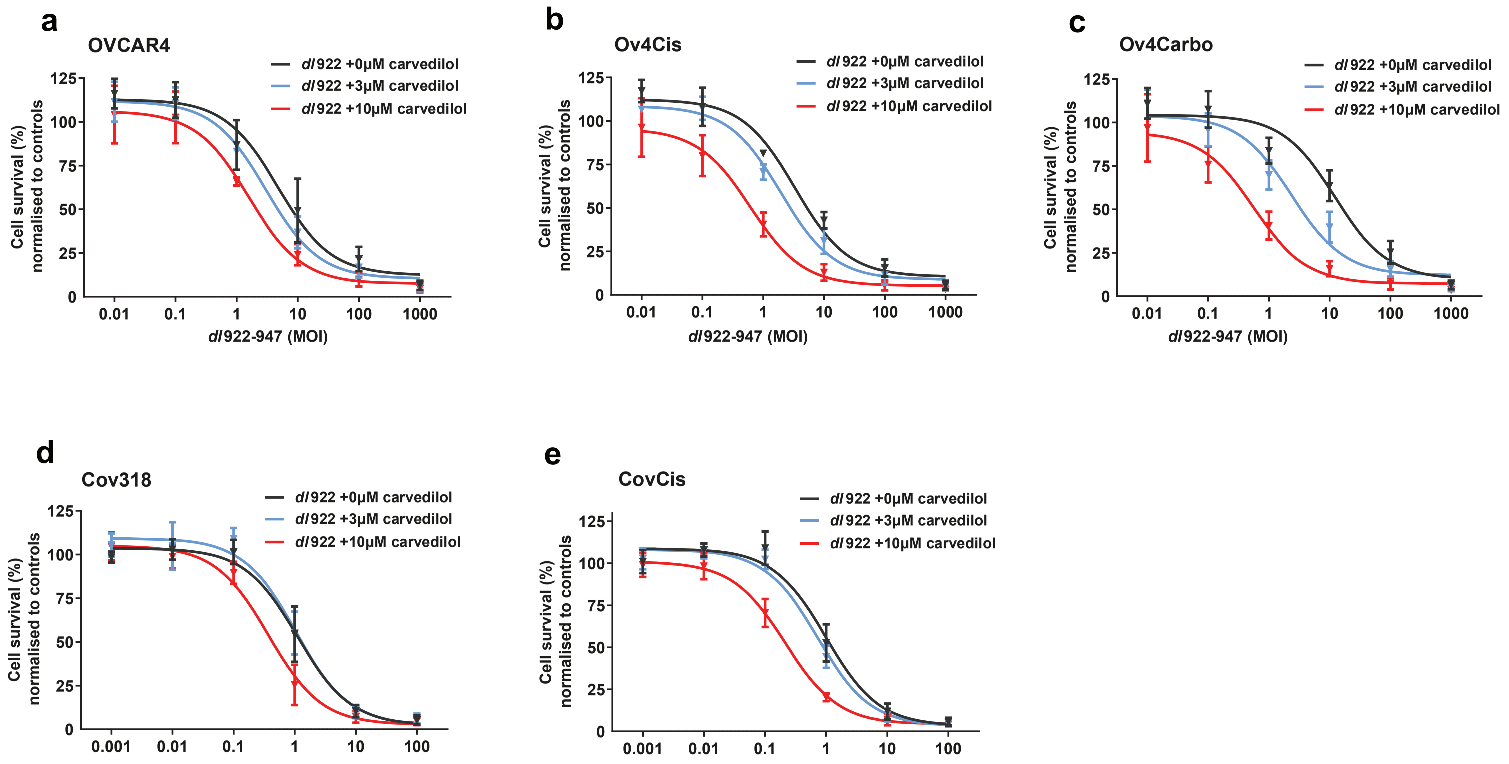

Supplementary Figure 1. **Carvedilol synergises with *dl*922-947 in HGSC cell lines.**
Representative ATP-based viability (CTG) survival curves from single biological repeat experiments are shown in **a)** OVCAR4, **b)** cisplatin-resistant Ov4Cis and **c)** carboplatin-resistant Ov4Carbo cell lines treated with *dl*922-947 and carvedilol (3-10μM). Mean ± SEM for three technical repeats are shown. These experiments are also shown as individual data points in Figure 1a.
Representative ATP-based viability (CTG) survival curves from single biological repeat experiments are shown in **d)** Cov318, and **e)** cisplatin-resistant CovCis cells lines treated with *dl*922-947 and carvedilol (3-10μM). Mean ± SEM for three technical repeats are shown. These experiments are also shown as individual data points in Figure 1b.

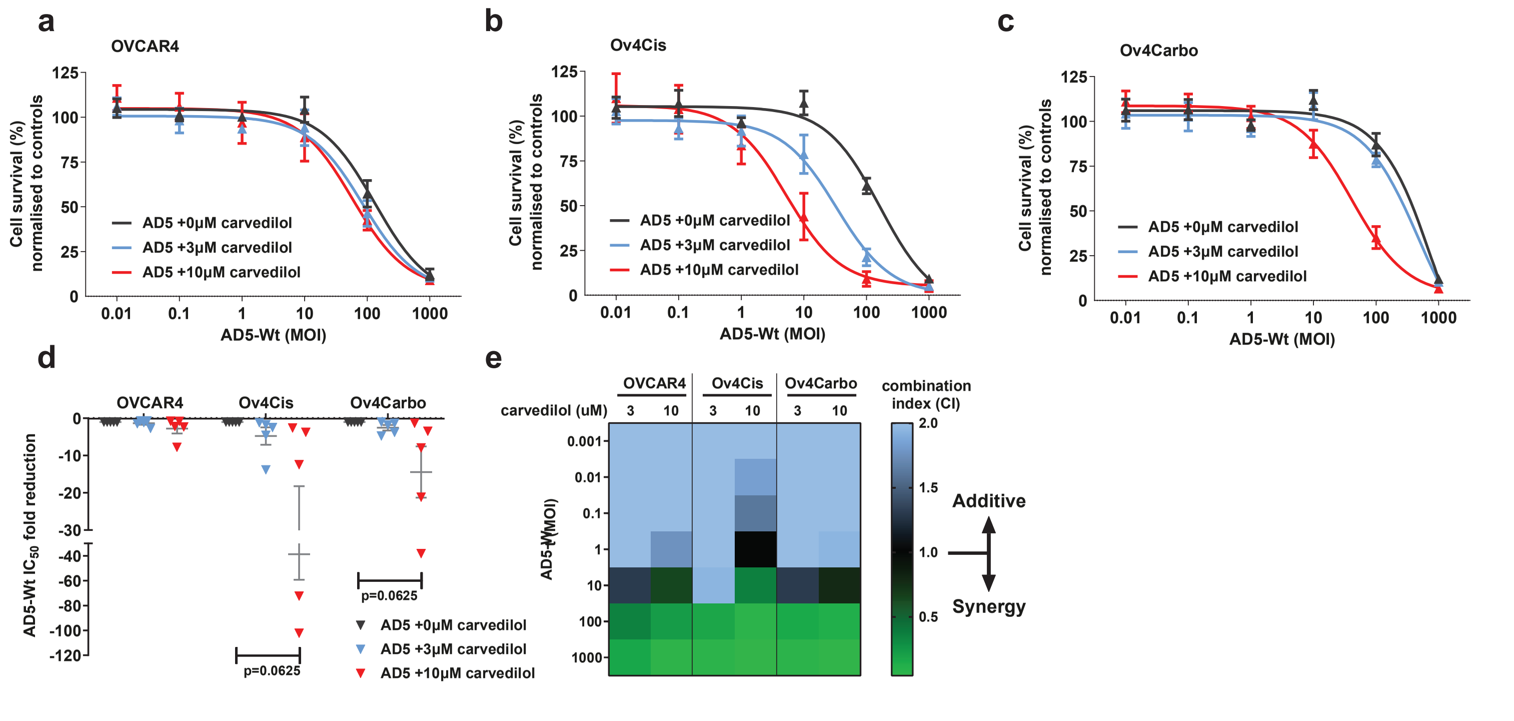

Supplementary Figure 2. **Carvedilol synergises with wildtype AD5 in platinum-resistant HGSC in vitro.**
Representative ATP-based viability (CTG) survival curves in **(a)** OVCAR4, **(b)** cisplatin-resistant Ov4Cis, **(c)** carboplatin-resistant Ov4Carbo cells lines treated with wild-type AD5 and carvedilol (3-10µM). **(d)** Fold reductions of wild-type AD5 IC50 in CTG assays following carvedilol treatment compared to virus-alone in the OVCAR4 cell line panel (n= 5 biological repeats; mean ±SEM). **(e)** Heatmap of the combination indexes between carvedilol and wildtype AD5 calculated from ATP-based viability assays

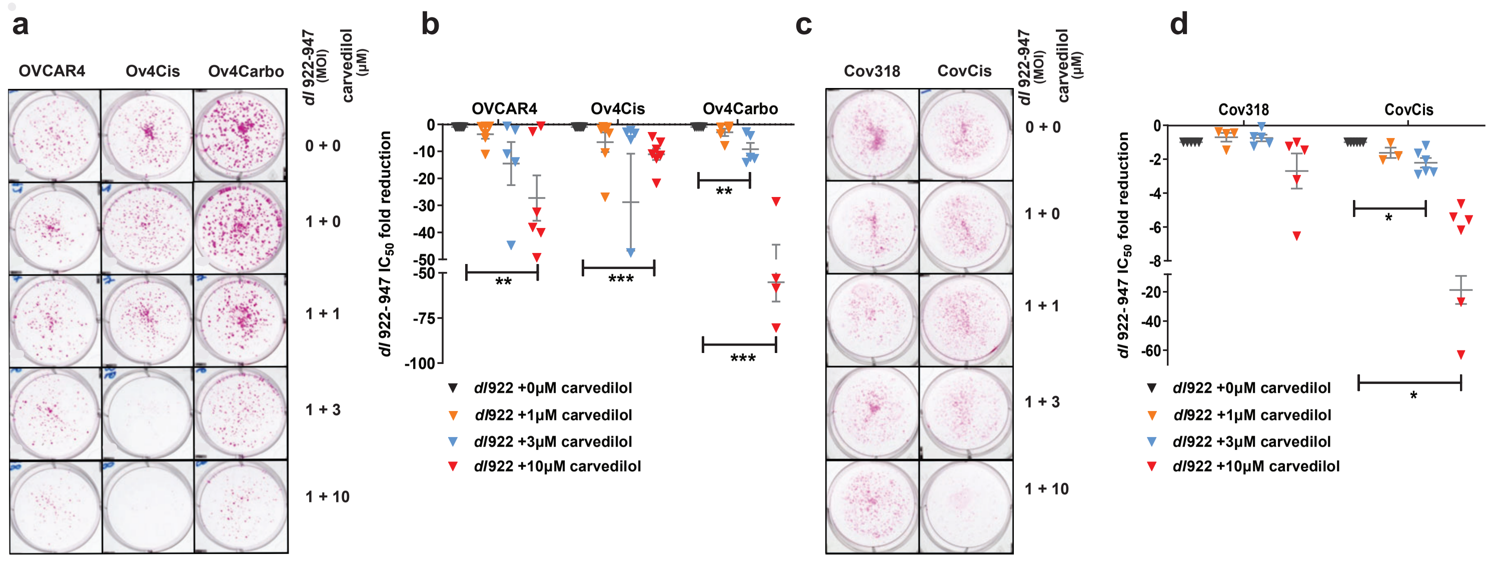

Supplementary Figure 3. **Colony formation assays confirm synergy between carvedilol and *dl*922-947.**
(a) Representative images of colony formation assays in the OVCAR4 cell line panel treated with *dl*922-947 and carvedilol (1-10μM). **(b)** Fold reductions of ***dl***922-947 IC50 in colony formation assays following carvedilol treatment compared to virus-alone in the OVCAR4 panel (OVCAR4: n=6; Ov4Cis n=7; Ov4Carbo n=5 biological repeats). **(c)** Representative images of colony formation assays in the COV318 cell line panel treated with ***dl***922-947 and carvedilol (1-10μM). **(d)** Fold reductions of ***dl***922-947 IC50 in colony formation assays following carvedilol treatment compared to virus-alone in the COV318 panel (COV318: n=5, COVcis: n=6 biological repeats). Mean ±SEM; Wilcoxon matched-pairs signed rank test, * P ≤0.05; ** P ≤0.01, *** P ≤0.001).

a.

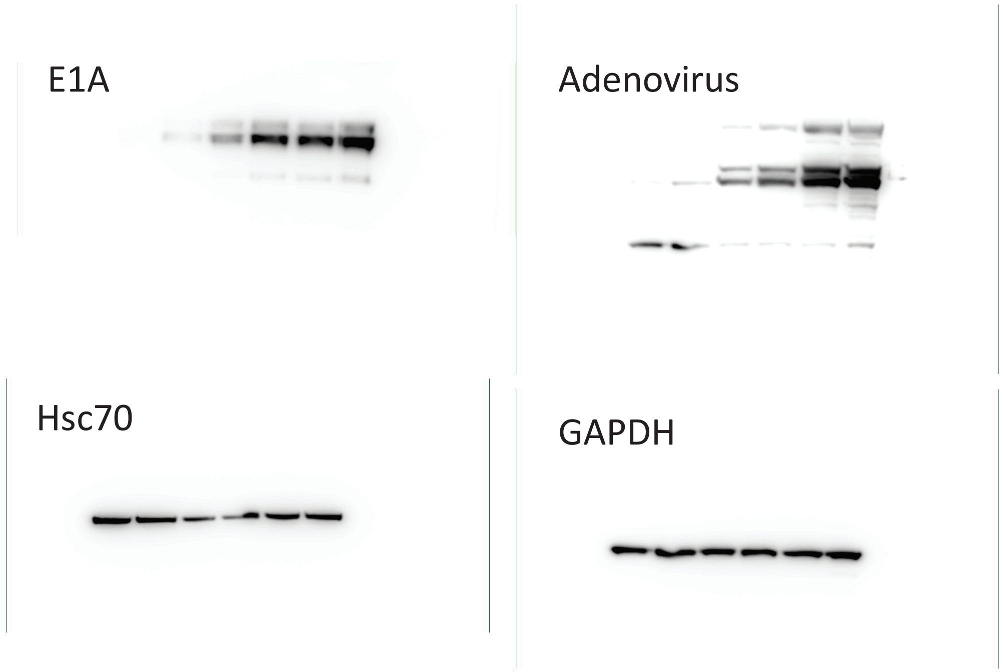

b.

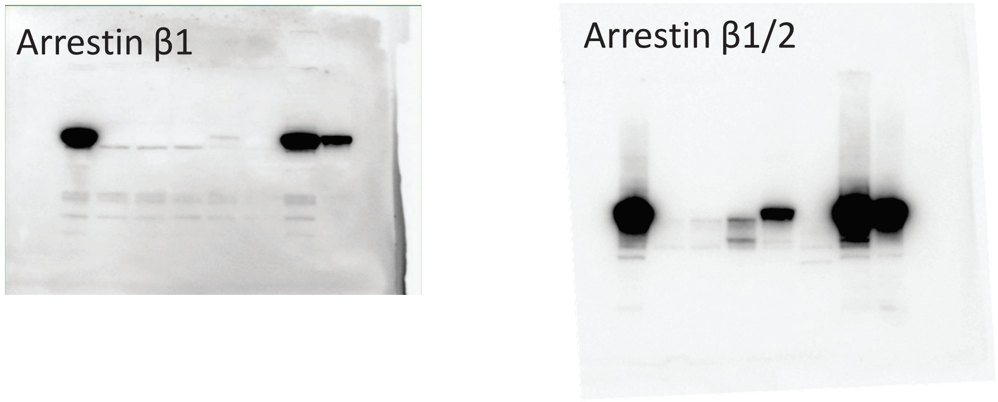

c.

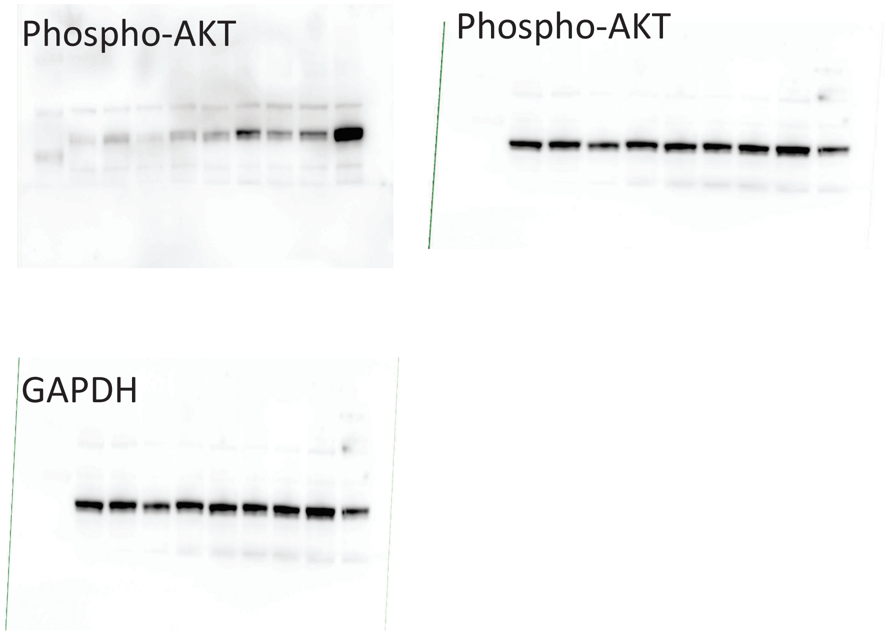

Supplementary Figure 4

a. Uncropped westerns blots from Figure 2c
b. Uncropped western blots from Figure 3e
   Maximum contrast is shown
c. Uncropped western blots from Figure 4a

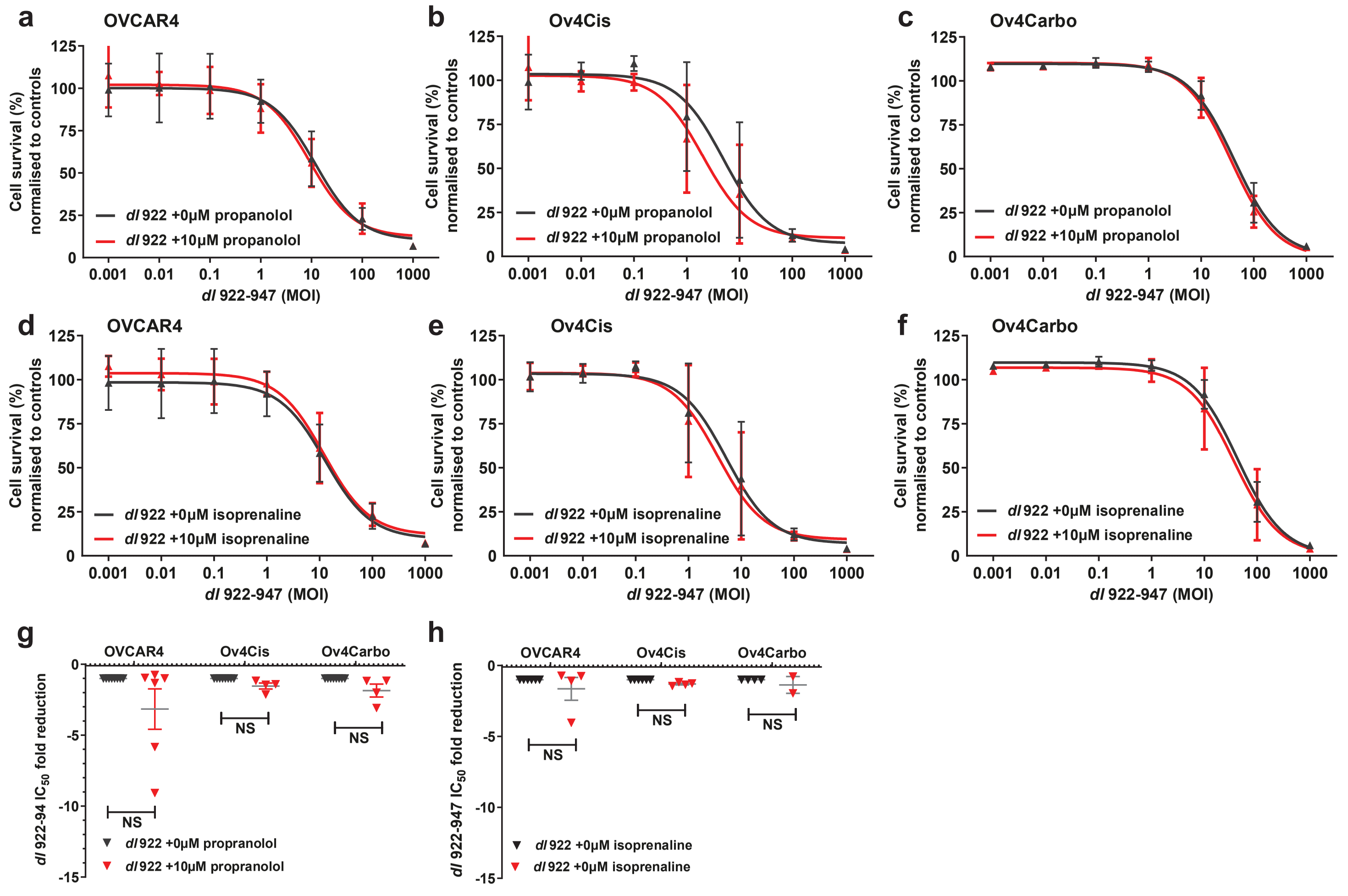

Supplementary Figure 5. Classical β-blockers and agonists do not synergise with *dl*922-947.
Representative ATP-based viability (CTG) survival curves in **(a)** OVCAR4, **(b)** cisplatin-resistant Ov4Cis, **(c)** carboplatin-resistant Ov4Carbo cells lines treated with ***dl***922-947 and propranolol (10µM). Representative ATP-based viability (CTG) survival curves in **(d)** OVCAR4, **(e)** cisplatin-resistant Ov4Cis, **(f)** carboplatin-resistant Ov4Carbo cells lines treated with ***dl***922-947 and isoprenaline (10µM). Fold reductions of ***dl***922-947 IC50 in CTG assays following carvedilol treatments compared to virus-alone in the OVCAR4 cell line panel with **(g)** propranolol (n= 5 biological repeats; mean ±SEM; ns P >0.05) and **(h)** isoprenaline (n= 3 biological repeats; mean ±SEM; ns P >0.05).

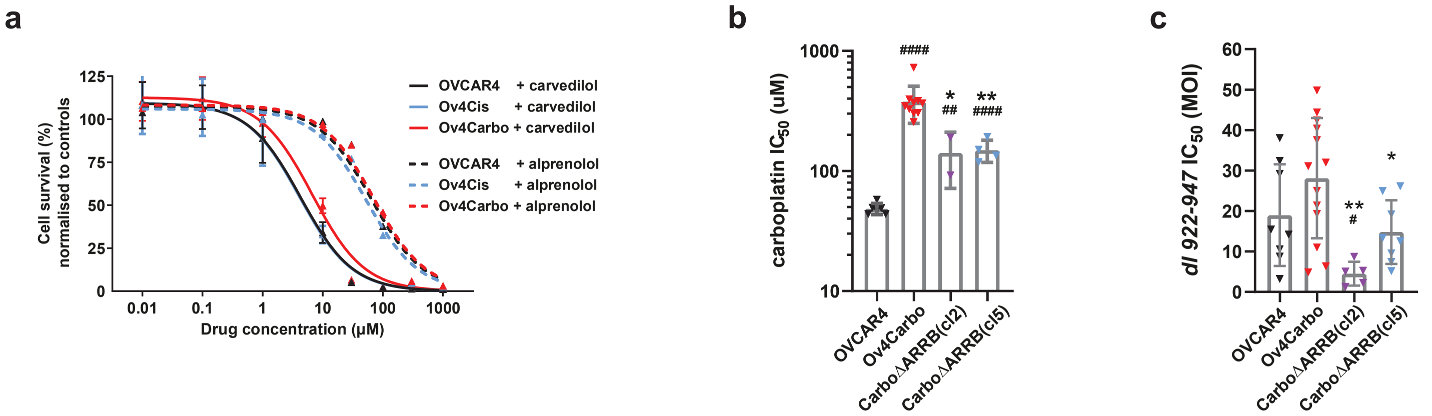

Supplemental Figure 6. Alprenolol dose response curves and IC50 data in β-arrestin CRISPR clones.
(a) Representative ATP-based viability assays (CTG) of carvedilol (solid lines) and alprenolol (dashed lines) dose responses in the OVCAR4 cell line panel. **(b)** Carboplatin IC50 in the OVCAR4 β-arrestin-knockout panel (OVCAR: n=6; Ov4Carbo: n=10; ΔARRB(cl2): n=2; ΔARRB(cl2): n=4 biological repeats; mean ± SD; unpaired T-test, # relative to OVCAR4, * relative to Ov4Carbo, * P ≤0.05; ** and ## P ≤0.01, *** and ### P ≤0.001; #### P ≤0.0001). **(c)** *dl*922-947 IC50 in the OVCAR4 β-arrestin-knockout panel (OVCAR: n=8; Ov4Carbo: n=13; ΔARRB(cl2): n=5; ΔARRB(cl2): n=8 biological repeats; mean ± SD; unpaired t-test, # relative to OVCAR4 and * relative to Ov4Carbo, * P ≤0.05; ** P ≤0.01)
