# Supplementary material for: Carvedilol targets β-arrestins to rewire innate immunity and improve oncolytic adenoviral therapy
Source: Commun Biol. 2022 Feb 3;5:106. doi: 10.1038/s42003-022-03041-4 (PMC8813932; doi:10.1038/s42003-022-03041-4)
Supplement: Supplementary file 3 — Description of Additional Supplementary Files [file 42003_2022_3041_MOESM3_ESM.pdf]

Description of Additional Supplementary Files

File name: Supplementary Data 1

Description: Log2-surviving fraction of the drug screen in cisplatin-resistant HGSC. Complete list of log2-surviving fraction for each well of the compound library screen in OVCAR4 and Ov4Cis cells. Results for all experimental conditions are shown including control-treated wells. log2-surviving fraction (s.f) is shown following treatment of OVCAR4 and Ov4Cis with the indicated drugs. Library = s.f. in wells treated with compound library drug compared to vehicle treatment. Library + adenovirus = s.f. in wells treated with compound library drug + adenovirus compared to adenovirus alone. A color gradient was applied to s.f. scores for visualisation purposes (gradient minimum (red): -6; midpoint (white): 0; maximum (green): 6).

File name: Supplementary Data 2

Description: Log2-surviving fraction of the drug screen in carboplatin-resistant HGSC. Complete list of log2-surviving fraction for each well of the compound library screen in OVCAR4 and Ov4Carbo cells. Results for all experimental conditions are shown including control-treated wells. log2-surviving fraction (s.f) is shown following treatment of OVCAR4 and Ov4Cis with the indicated drugs. Library = s.f. in wells treated with compound library drug compared to vehicle treatment. Library + adenovirus = s.f. in wells treated with compound library drug + adenovirus compared to adenovirus alone. A color gradient was applied to s.f. scores for visualisation purposes (gradient minimum (red): -6; midpoint (white): 0; maximum (green): 6).

File name: Supplementary Data 3

Description: Hit drugs identified in the both compound library screens comparing OVCAR4 to Ov4Cis and also OVCAR4 to Ov4Carbo. log2-surviving fraction (s.f) is shown following treatment with the indicated drugs. Library = surviving fraction (s.f.) in wells treated with compound library drug compared to vehicle treatment. Library + dl922-947 = s.f. in wells treated with compound library drug + dl922-947 compared to dl922-947 alone. A color gradient was applied to s.f. scores for visualisation purposes (gradient minimum (red): -6; midpoint (white): 0; maximum (green): 6).

File name: Supplementary Data 4

Description: β-blocker and α-blocker drug log2-surviving fraction included in the compound library screens. log2-surviving fraction (s.f) is shown following treatment of OVCAR4, Ov4Cis and Ov4Carbo with the indicated drugs. Library = s.f. in wells treated with compound library drug compared to vehicle treatment. Library + adenovirus = s.f. in wells treated with compound library drug + adenovirus compared to adenovirus alone. For the purposes of this study, a compound was considered as a hit if s.f. < -2 only for library + adenovirus treatments. A color gradient was applied to s.f. scores for visualisation purposes (gradient minimum (red): -6; midpoint (white): 0; maximum (green): 6).
