# Supplementary material for: Carvedilol targets β-arrestins to rewire innate immunity and improve oncolytic adenoviral therapy
Source: Commun Biol. 2022 Feb 3;5:106. doi: 10.1038/s42003-022-03041-4 (PMC8813932; doi:10.1038/s42003-022-03041-4)
Supplement: Supplementary file 8 — Reporting Summary [file 42003_2022_3041_MOESM8_ESM.pdf]

nature portfolio

Corresponding author(s): Lockley
Last updated by author(s): Dec 10, 2021

Reporting Summary

Nature Portfolio wishes to improve the reproducibility of the work that we publish. This form provides structure for consistency and transparency in reporting. For further information on Nature Portfolio policies, see our Editorial Policies and the Editorial Policy Checklist.

Statistics

For all statistical analyses, confirm that the following items are present in the figure legend, table legend, main text, or Methods section.

n/a	Confirmed	
☐	☒	The exact sample size (*n*) for each experimental group/condition, given as a discrete number and unit of measurement
☐	☒	A statement on whether measurements were taken from distinct samples or whether the same sample was measured repeatedly
☐	☒	The statistical test(s) used AND whether they are one- or two-sided<br>*Only common tests should be described solely by name; describe more complex techniques in the Methods section.*
☒	☐	A description of all covariates tested
☐	☒	A description of any assumptions or corrections, such as tests of normality and adjustment for multiple comparisons
☐	☒	A full description of the statistical parameters including central tendency (e.g. means) or other basic estimates (e.g. regression coefficient) AND variation (e.g. standard deviation) or associated estimates of uncertainty (e.g. confidence intervals)
☐	☒	For null hypothesis testing, the test statistic (e.g. *F*, *t*, *r*) with confidence intervals, effect sizes, degrees of freedom and *P* value noted<br>*Give P values as exact values whenever suitable.*
☒	☐	For Bayesian analysis, information on the choice of priors and Markov chain Monte Carlo settings
☒	☐	For hierarchical and complex designs, identification of the appropriate level for tests and full reporting of outcomes
☒	☐	Estimates of effect sizes (e.g. Cohen's *d*, Pearson's *r*), indicating how they were calculated

Our web collection on statistics for biologists contains articles on many of the points above.

Software and code

Policy information about availability of computer code

Data collection	No software was used
Data analysis	GraphPad Prismv.8.0 was used for statistical analysis. Discovery workbench v.4.0 was used for Mesoscale analysis. CompuSyn v.1 was used for combination index experiments. All are detailed in the Methods section of the manuscript

For manuscripts utilizing custom algorithms or software that are central to the research but not yet described in published literature, software must be made available to editors and reviewers. We strongly encourage code deposition in a community repository (e.g. GitHub). See the Nature Portfolio guidelines for submitting code & software for further information.

Data

Policy information about availability of data

All manuscripts must include a data availability statement. This statement should provide the following information, where applicable:

- Accession codes, unique identifiers, or web links for publicly available datasets
- A description of any restrictions on data availability
- For clinical datasets or third party data, please ensure that the statement adheres to our policy

Complete data from the two library screens are currently presented as Supplementary Data 1 and 2. These data will are now available via Figshare : (https://figshare.com/articles/figure/Carvedilol_targets_arrestins_to_rewire_innate_immunity_and_improve_oncolytic_adenoviral_therapy/17013317). This reference is provided in the data availability statement in the final manuscript

Field-specific reporting

Please select the one below that is the best fit for your research. If you are not sure, read the appropriate sections before making your selection.

☒ Life sciences ☐ Behavioural & social sciences ☐ Ecological, evolutionary & environmental sciences

For a reference copy of the document with all sections, see nature.com/documents/nr-reporting-summary-flat.pdf

Life sciences study design

All studies must disclose on these points even when the disclosure is negative.

Sample size	Sample size for animal experiments was calculated using 'sample size-survival analysis' software www.sample-size.net
Data exclusions	Mice were excluded if they did not exhibit an average radiance between 10^5 - 10^7 p/s/cm2/sr by day 21 post inoculation and 34 mice excluded on this basis from a total of 152.
Replication	The established scientific standard of at lest 3 biological repeats was applied throughout. All attempts at replication are included in the statistical analysis
Randomization	Mice were randomly allocated to treatment groups
Blinding	Researchers were blinded to the treatment groups for all animal experiments

Reporting for specific materials, systems and methods

We require information from authors about some types of materials, experimental systems and methods used in many studies. Here, indicate whether each material, system or method listed is relevant to your study. If you are not sure if a list item applies to your research, read the appropriate section before selecting a response.

Materials & experimental systems

n/a	Involved in the study	
☐	☒	Antibodies
☐	☒	Eukaryotic cell lines
☒	☐	Palaeontology and archaeology
☐	☒	Animals and other organisms
☒	☐	Human research participants
☒	☐	Clinical data
☒	☐	Dual use research of concern

Methods

n/a	Involved in the study	
☒	☐	ChIP-seq
☒	☐	Flow cytometry
☒	☐	MRI-based neuroimaging

Antibodies

Antibodies used	The following antibodies were used:<br>E1A: Santa Cruz sc-430, 1:1000; Adenovirus: Abcam ab36851, 1:5000; Hsc70: Abcam ab36851, 1:10000; GAPDH: Santa Cruz sc-47724, 1:10000; phospho-Akt: Cell Signalling 9271L, 1:1000; Akt: Cell Signalling 9272; phospho-ERK1/2: Cell Signalling 4370, 1:1000; ERK1/2: Cell Signalling 9102; ARRB1/2: Cell Signalling 4674S, 1:500; ARRB1: Cell Signalling 12697S, 1:500. PAX8: Abcam ab13611, 1:100; F4/80: Biorad MCA497GA, 1:1000; Adenovirus: Abcam ab8251, 1:1000; DX5: Abcam ab133557, 1:250.
Validation	All antibodies were used in accordance with the manufacturer's website

Eukaryotic cell lines

Policy information about cell lines

Cell line source(s)	Cell lines were obtained from collaborators
Authentication	All cell lines underwent 16 locus STR verification (DNA Diagnostics Centre, London, UK: June 2015-February 2016 and European Collection of Authenticated Cell Lines August 2019)
Mycoplasma contamination	All cell lines were tested weekly for mycoplasma contamination and all cell lines used were mycoplasma negative
Commonly misidentified lines<br>(See ICLAC register)	N/A

Animals and other organisms

Policy information about studies involving animals; ARRIVE guidelines recommended for reporting animal research

Laboratory animals	This study used 6-week old female CD1nu/nu mice
Wild animals	N/A
Field-collected samples	N/A
Ethics oversight	Experiments were conducted following Institutional Review Board Approval and under UK government Home Office license (P1EE3ECB4)

Note that full information on the approval of the study protocol must also be provided in the manuscript.
